# A PBPK-VBE approach for bioequivalence extrapolation of senaparib capsule strengths in the context of nonlinear pharmacokinetics: bridging from the clinical starting dose to the linear pharmacokinetic range

**DOI:** 10.3389/fphar.2026.1918344

**Published:** 2026-09-04

**Authors:** Xiaofei Wu, Ziyang Wang, Yanna He, Keheng Wu, Bo Liu, Ning Ma, Suixiong Cai, Cong Xu, Yanhua Xu, Shiqing Zhao, Hongyun Wang

**Affiliations:** 1 Clinical Pharmacology Research Center, Peking Union Medical College Hospital, Beijing Key Laboratory of Key Technologies for Early Clinical Trial Evaluation Innovative Drugs for Major Diseases, Chinese Academy of Medical Sciences & Peking Union Medical College, Beijing, China; 2 Shanghai Impact Therapeutics Co., Ltd., Shanghai, China; 3 Yinghan Pharmaceutical Technology (Shanghai) Co., Ltd., Shanghai, China; 4 School of Chemistry and Chemical Engineering, Shanghai University of Engineering Science, Shanghai, China

**Keywords:** formulation bridge, PARP inhibitor, PBPK model, senaparib, VBE

## Abstract

**Aims:**

Senaparib (Sepalna®), a novel oral poly (adenosine diphosphate -ribose) polymerase (PARP) 1/2 inhibitor, was approved in China in 2025 as maintenance therapy for advanced ovarian cancer. The recommended starting dose is 100 mg once daily, which may be adjusted to 80 mg, 60 mg, or 40 mg to manage adverse events. While conventional bioequivalence (BE) clinical studies established equivalence between the 10 mg and 20 mg capsules strengths at the 100 mg dose level, these findings cannot be extrapolated to the lower dose range due to senaparib’s nonlinear pharmacokinetics and the current absence of definitive regulatory guidance for such scenarios. This study aimed to evaluate the bioequivalence between the 10 mg and 20 mg capsule strengths of senaparib, a drug exhibiting nonlinear pharmacokinetics, across the 20–80 mg dose range.

**Methods:**

We developed and validated an integrated approach combining physiologically based pharmacokinetic (PBPK) modeling with *in vitro* dissolution data to evaluate virtual bioequivalence (VBE) between the two capsule strengths across the 20–80 mg dose range.

**Results:**

Under all evaluated conditions including variations in sample size, intra-individual variability levels, and prandial states (fasted and fed), the geometric mean ratios (GMRs) of key pharmacokinetic (PK) parameters consistently approached 100%, thereby confirming bioequivalence across the specified dosing range.

**Conclusion:**

This PBPK-VBE framework provides supportive evidence for bioequivalence bridging of senaparib capsule strengths across the linear dose range, illustrating the potential utility of this approach for formulation bridging when direct clinical BE studies across all dose levels are impractical.

## 1 Introduction

Senaparib (IMP4297) is a novel, orally administered, highly potent, and selective poly (ADP-ribose) polymerase 1/2 (PARP1/2) independently developed by Shanghai Impact Therapeutics Co., Ltd. It exerts its antitumor effect through synthetic lethality, inducing cell death in tumor cells with defective DNA damage repair by inhibiting the DNA single-strand break repair pathway (Gao et al., 2023; Wu et al., 2024; Cai et al., 2025). Preclinical studies have shown that senaparib possesses superior *in vivo* antitumor activity compared to other PARP inhibitors such as Olaparib and Niraparib (Cai et al., 2022; Cai et al., 2025). On 16 January 2025, senaparib (Sepalna®) was approved by China’s National Medical Products Administration (NMPA) as a monotherapy for the maintenance treatment of adult patients with advanced epithelial (FIGO stages III and IV) high-grade ovarian, fallopian tube, or primary peritoneal cancer who achieved a complete or partial response to first-line platinum-based chemotherapy (National Medical Products Administration, 2025). The recommended starting dose is 100 mg once daily, orally administered without food (Wu et al., 2024). Dose adjustments were implemented for the management of adverse events (AEs). Adjustments were to be performed in a stepwise manner——one dose level per reduction——with a maximum of three reductions allowed (to 80 mg, 60 mg, or 40 mg) (IMPACT Therapeutics, 2025).

The 10 mg senaparib capsule was evaluated in the pivotal clinical trials. However, to enhance patient medication adherence, we intend to introduce an additional 20 mg capsule, supplementing the existing 10 mg formulation. Consequently, establishing bioequivalence (BE) between these two strengths became a critical requirement for the new drug application (NDA). BE studies (IMP4297-110-1 and IMP4297-110-2) successfully demonstrated that a 100 mg dose administered as five 20 mg capsules was bioequivalent to ten 10 mg capsules in the fasted and fed states. However, two phase I dose-escalation studies (IMP4297-2016-AU01, IMP4297-2016-CN01) conducted in patients with advanced solid tumors revealed that pharmacokinetic (PK) exposure—specifically, the area under the concentration-time curve (AUC) and maximum plasma concentration (C_max_)—increased dose-proportionally within the 20–80 mg range but plateaued at doses of 80 mg and above (80–150 mg) (Gao et al., 2023; Cao et al., 2023). This saturation in exposure indicated a nonlinear absorption process. According to current regulatory guidelines such as the International Council for Harmonisation of Technical Requirements for Pharmaceuticals for Human Use (ICH) M13A on “Bioequivalence for Immediate-Release Solid Oral Dosage Forms,” BE should be established at the lowest strength for drugs exhibiting a less-than-dose-proportional increase in AUC and/or C_max_ across the therapeutic range when attributable to absorption saturation. Nevertheless, existing guidelines lack explicit recommendations on a critical issue: which doses should be selected to demonstrate BE for nonlinear pharmacokinetic compounds following changes in dosage strength. Consequently, the BE conclusion established at the clinical dose (100 mg) cannot be extrapolated to lower adjusted doses (e.g., 40 mg, 60 mg, and 80 mg).

Virtual Bioequivalence (VBE) presents a promising solution to such challenges, offering advantages such as low cost, short timelines, and scientific transparency (Alotaiq and Dermawan, 2024; Kollipara et al., 2024). For example, during the 2018 U.S. Food and Drug Administration (FDA) approval of Elagolix tablets, a physiologically based pharmacokinetic (PBPK) model incorporating *in vitro* dissolution data was developed to establish clinically relevant dissolution criteria, supporting a biowaiver for additional clinical BE studies between commercial and clinical trial batches (Mukherjee et al., 2023). Similarly, a PBPK-VBE approach successfully demonstrated BE for a formulation change of SM2 tablets, thereby supporting a waiver of the requirement for a traditional *in vivo* BE study (Karnati et al., 2023). These successful applications not only affirm the reliability of PBPK models but also offer adaptable regulatory strategies that can facilitate innovative drug development.

To address the BE challenge posed by the strength change of senaparib—a drug with nonlinear pharmacokinetics—this study developed and validated an integrated approach combining PBPK modeling with *in vitro* dissolution data. The BE analysis was systematically conducted between the two capsule strengths across the linear dose range under various conditions, including different sample sizes, levels of intra-individual variability, and fasted/fed states.

## 2 Materials and methods

### 2.1 Overall strategy

**FIGURE 1:**
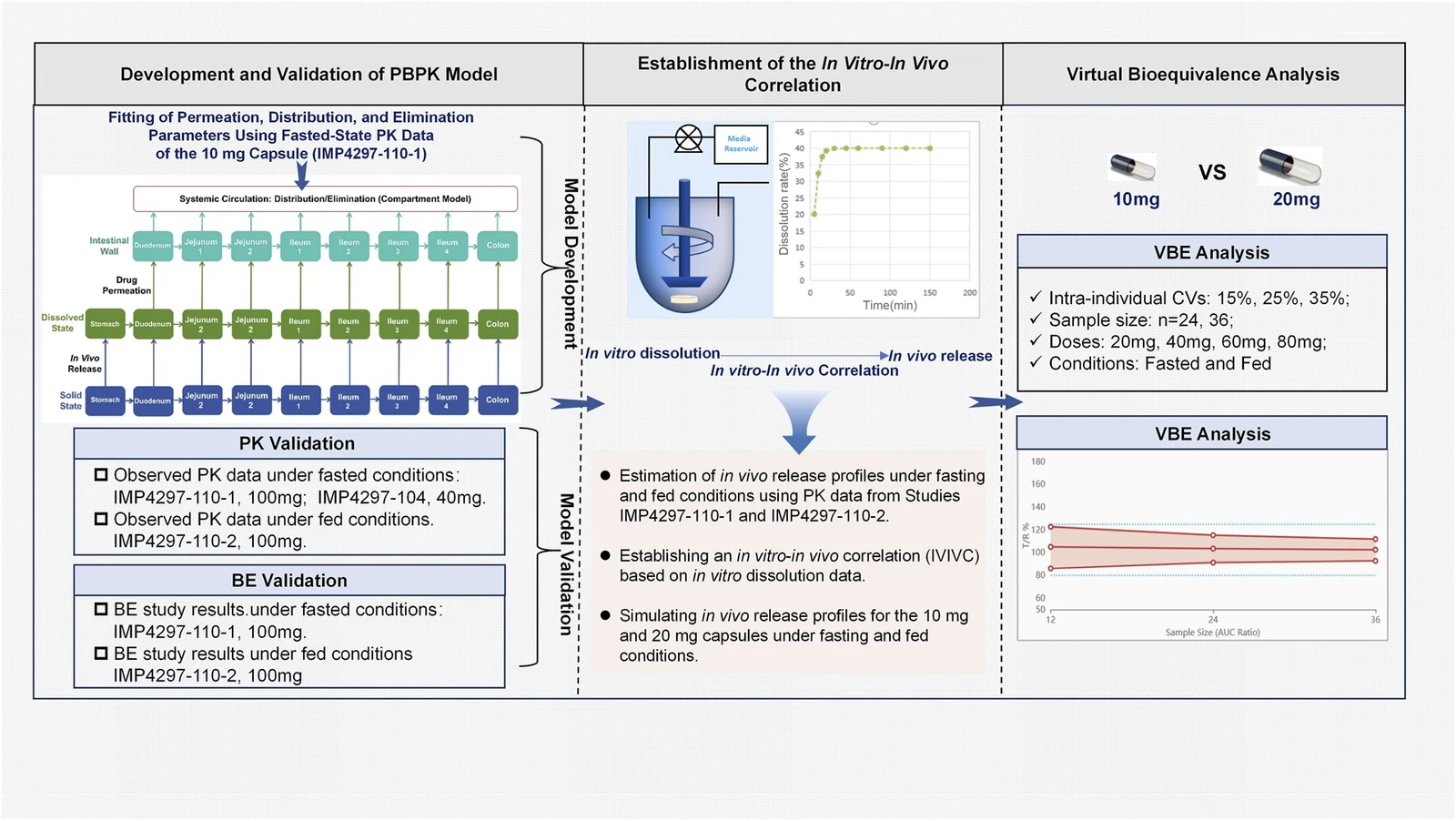
The overall framework of PBPK-VBE approach.

The overall framework of the PBPK-VBE approach, as illustrated in Figure 1, consisted of the following steps: (1) Estimation of gastrointestinal (GI) PBPK model parameters for permeation, distribution, and elimination, as well as *in vivo* release profiles, using observed PK data; (2) Establishment of the *in vitro*-*in vivo* correlation (IVIVC) between *in vitro* dissolution data and *in vivo* release profiles; (3) Simulation of the absorption processes and PK characteristics of senaparib using the PBPK model; (4) Conducting virtual BE studies to evaluate the potential differences in PK characteristics among different formulations containing identical active pharmaceutical ingredient(API).

The PBPK-VBE analysis was performed using B2O Simulator (version 3.0, Yinghan Pharmaceutical Technology (Shanghai) Co., Ltd.). In this PBPK model, the GI tract was divided into nine anatomical compartments, including one stomach, one duodenum, two jejunum, four ileum and one colon. The model incorporated physiological mechanisms such as gastric emptying and intestinal motility, regional gastrointestinal blood flow, and relative permeability, enabling the simultaneous simulation of drug transit, release, permeation, and plasma concentration-time changes (Zhang et al., 2021). The distribution and elimination of the drug following its entry into the systemic circulation were characterized by compartmental models.

### 2.2 Study data

#### 2.2.1 Clinical study

IMP4297-110-1 and IMP4297-110-2 were randomized, single-dose, two-period, two-sequence crossover BE studies conducted in healthy Chinese participants. Study IMP4297-110-1 evaluated the PK of a 100 mg dose of senaparib administered as either five 20 mg capsules or ten 10 mg capsules in the fasted state. Study IMP4297-110-2 evaluated the same two formulations administered as a 100 mg dose in the fed state. The PK data from study IMP4297-110-1 were used to estimate the permeation, distribution and elimination parameters of senaparib, validate the *in vitro* fasted-state dissolution method, establish the IVIVC, and verify the model’s simulation performance in the fasted state. The PK data from study IMP4297-110-2 were used to validate the *in vitro* fed-state dissolution method, establish the corresponding IVIVC, and verify the model’s simulation performance in the fed state. The BE results for studies IMP4297-110-1 and IMP4297-110-2 were summarized in Supplementary Table S1. The 90% CIs for the GMRs of AUC_0-last_, AUC_0-inf_, and C_max_ between the two strengths of senaparib fell within the acceptance range (80.00%–125.00%). Collectively, these results demonstrated bioequivalence at the 100 mg dose level between the two strengths in both fasted and fed states.

IMP4297-104 was a Phase I, open-label, fixed-sequence study conducted in healthy participants to evaluate the effects of multiple-dose itraconazole or rifampicin on the PK of a single-dose senaparib capsule (Hu et al., 2023). The PK data of 40 mg senaparib monotherapy from Period 1 were used to evaluate the model’s predictive performance across the linear dose range (20–80 mg). Further clinical study details were provided in Supplementary Text S1.

#### 2.2.2 *In vitro* dissolution profile

Biorelevant media—fasted-state simulated intestinal fluid (FaSSIF, pH = 6.5) and fed-state simulated intestinal fluid (FeSSIF, pH = 5.0)—were selected to mimic the physiological dissolution environment. Accordingly, *in vitro* dissolution testing of senaparib (one 20 mg capsule and two 10 mg capsules) was conducted in these two biorelevant media. The experiment was conducted at the temperature of 37 °C ± 0.5 °C and the speed of 50 r/min with 900 mL of dissolution medium according to the paddle method in Chinese Pharmacopoeia Commission (2020). The samples (10 mL) were withdrawn at 5, 10, 15, 20, 30, 45, 60, 90, 120, and 150 min and an equivalent volume of fresh medium was added in time. The collected samples were filtrated and detected by a validated high-performance liquid chromatography (HPLC) method. All experiments were performed in twelve replicates.

### 2.3 Development and validation of the fasted-state PBPK model

#### 2.3.1 Estimation of permeation, distribution, and elimination parameters

The effective permeability coefficient (P_eff_), apparent volume of distribution (V_d_), elimination rate constant from the central compartment to the peripheral compartment(k_12_), elimination rate constant from the peripheral compartment to the central compartment(k_21_), clearance (CL), and inter-individual variability were estimated within the compartment model using the standard two-stage approach (STS), based on the individual PK data from the 10 mg senaparib capsule in clinical study IMP4297-110-1. The STS approach enables estimation of individual PK parameters followed by derivation of typical population values and inter-individual variability, which can be incorporated into virtual population simulations. The rate constants k_12_ and k_21_ were required only when a two-compartment model was selected.

During PBPK model development, a fundamental assumption was proposed that the parameters of permeation, distribution, and elimination remained invariant across capsule strengths, doses, and prandial states. Consequently, the *in vivo* PK variability was attributed solely to differences in dissolution profiles. Once the model parameters estimated from the PK observations in the fasted state were validated, they could be applied to simulate various scenarios involving capsule strengths, doses, and prandial states, with only the corresponding *in vivo* release data being adjusted.

#### 2.3.2 Establishment of the *in vitro*-*in vivo* correlation in the fasted state

The *in vivo* release profile was fitted based on PK data from study IMP4297-110-1, characterized by a Weibull function (shown in Equation 1). The fitted *in vivo* release profile was then compared with the *in vitro* dissolution data to evaluate their correlation and linearity, thereby establishing the IVIVC for converting *in vitro* dissolution data to *in vivo* release behavior.

$$F\left(t,kd,\beta ,tlag\right)=F_{\max}*\left(1-e^{-kd*{\left(t-tlag\right)}^{\beta }}\right)$$ (1)

Note: *F*_max_ represents the maximum dissolution rate, *t* represents time, *k*_*d*_ represents the dissolution rate constant, *β* represents the shape factor, and *t*_*lag*_ represents the delayed dissolution time.

#### 2.3.3 Validation of the fasted-state PBPK model

To verify the reliability of the fasted-state PBPK model, the *in vivo* release was estimated based on the *in vitro* dissolution data of the two formulations (10 mg and 20 mg) in the fasted state. Subsequently, the PK profiles were simulated in a virtual healthy population and compared with the observed data from study IMP4297-110-1 (100 mg dose, fasted-state). Agreement between the simulated and observed data verified the reliability of the fasted-state PBPK model.

Subsequently, the model’s capability for dose extrapolation to the linear dose range (20–80 mg) was evaluated. The fasted-state PBPK model was used to simulate PK characteristics at a dose of 40 mg, and the results were compared with the observed PK data from study IMP4297-104 following administration of a 40 mg dose (four 10 mg capsules). Agreement between the simulated and observed data at this lower dose confirmed the model’s capability to accurately simulate PK profiles across the linear dose range. For each dose level, quantitative verification was performed by calculating the predicted-to-observed (P/O) ratios for key PK parameters (C_max_ and AUC_0-last_). Prediction was considered acceptable if the ratios fell within the standard two-fold error range (0.5–2.0).

Moreover, to further verify the predictive performance of the fasted-state PBPK model for bioequivalence assessment, we performed the VBE analysis between the two formulations at a dose of 100 mg in the fasted state (five 20 mg capsules vs. ten 10 mg capsules). Simulations incorporated intra-individual coefficients of variation (CVs) of 15%, 25%, and 35%. Sample sizes of 24 and 36 participants were selected as representative VBE scenarios to cover relatively smaller and larger two-period crossover BE trial settings. The simulated VBE results were then compared with the observed results from study IMP4297-110-1.

#### 2.3.4 Sensitivity analysis of the fasted-state PBPK model

A sensitivity analysis was conducted to assess the robustness of the PBPK model for senaparib. Simulations were performed for a single 100 mg dose in the fasted state. Specifically, the analysis incorporated key parameters with a high potential to influence the C_max_ and AUC_0-inf_, including P_eff_, gastric solubility, intestinal solubility, V, and CL. For each selected parameter, a relative variation range of ±10% was applied. The sensitivity was evaluated by calculating the percentage change in predicted C_max_ and AUC_0-inf_ relative to the baseline.

### 2.4 Development and validation of the fed-state PBPK model

#### 2.4.1 Establishment of the *in vitro*-*in vivo* correlation in the fed state

Based on the assumptions described in Section 2.3.1, the permeation, distribution, and elimination parameters of the fed-state PBPK model were adopted from the fasted-state model. With these parameters fixed, the Weibull function parameters for *in vivo* release in the fed state were then fitted independently.

#### 2.4.2 Validation of the fed-state PBPK model

PK and VBE validation were performed following the development of the fed-state PBPK model. The simulated PK profiles and BE results were then compared with the observed data from study IMP4297-110-2 (100 mg dose, fed-state). Agreement between the simulated and observed data verified the reliability of the fed-state PBPK model. Moreover, quantitative verification was performed by calculating the predicted-to-observed (P/O) ratios for key PK parameters (C_max_ and AUC_0-last_). Prediction was considered acceptable if the ratios fell within the standard two-fold error range (0.5–2.0). However, due to the absence of low-dose clinical PK data for senaparib in the fed state, PK validation at lower doses was not performed.

### 2.5 VBE analysis across the linear dose range

VBE analyses were conducted to assess the bioequivalence between the test (20 mg capsule) and reference (10 mg capsule) formulations across the linear dose range (20, 40, 60, and 80 mg) in the fasted and fed states (Supplementary Text S2).

Inter-individual variability was characterized based on the fitting of PK parameters following administration of a 100 mg dose in the fasted state (IMP4297-110-1). Given that the observed intra-individual variability for senaparib in studies IMP4297-110-1 and IMP4297-110-2 ranged from 15.4% to 21.3%, the VBE analyses evaluated a wider range by incorporating intra-individual CVs of 15%, 25%, and 35%. These simulations were performed for sample sizes of 24 and 36 participants in the fasted and fed states.

Each simulation assessed PK exposures for different combinations of dissolution conditions, sample sizes, and intra-individual CVs. The BE results were determined by assessing whether the geometric mean ratios (GMRs) and their 90% confidence intervals (CIs) of the C_max_ and the AUC from time zero to the time of the last quantifiable concentration (AUC_0-last_) fell within the 80.00%–125.00% acceptance range.

## 3 Results

### 3.1 Development and validation of the fasted-state PBPK model

#### 3.1.1 Estimation of absorption, distribution, and elimination parameters

The permeation, distribution, and elimination parameters fitted in the fasted state were presented in Table 1. The comparison between the simulated profiles and observed PK data was shown in Supplementary Figure S1. The results demonstrated that the majority of observed data points in Supplementary Figure S1A fell within the 90% CIs of the simulated profiles. The mean of observed data closely aligned with the 50th percentile line of simulated data in Supplementary Figure S1B. These results indicated that the model fitted the data well and the current model parameters sufficiently characterized the observed PK profiles.

**TABLE 1:** Estimated permeation, distribution, and elimination parameters for senaparib capsules.

Permeation, distribution, and elimination parameters (Units)	Value
Gastric solubility (mg/L)	6.31
Intestinal solubility (mg/L)	0.05
P_eff_ (10^−4^ cm/s)	1.900
V (L/kg)	19.500
k_12_ (h^−1^)	0.175
k_21_ (h^−1^)	0.200
CL(L/h)	2.800
Intra-individual CVs of CL (%)	48

#### 3.1.2 Establishment of the *in vitro*-*in vivo* correlation in the fasted state

The *in vivo* release profiles were fitted based on PK observations from 10 mg to 20 mg capsules administered in the fasted state (Supplementary Table S2). The *in vitro* dissolution profiles of the two formulations in the fasted state were shown in Supplementary Table S3; Supplementary Figure S2. Subsequent linear correlation analysis between *in vitro* dissolution data and corresponding *in vivo* release profiles indicated a strong correlation within the first 20 min (R^2^ = 0.9749). However, significant divergence occurred subsequently (Supplementary Figure S3). This discrepancy may be attributed to the differences between *in vitro* and *in vivo* dissolution environments, causing a gradual decline in the *in vitro* dissolution rate. Nevertheless, the maximum dissolution and the dissolution profiles within the first 20 min were sufficient to characterize the *in vivo* release. Accordingly, Weibull function parameters were obtained by fitting the *in vitro* dissolution data from the first 20 min, enabling the simulation of the drug’s *in vivo* release in the fasted state (Table 2). The fitted curves closely matched the observed data (Supplementary Figure S4A-B).

**TABLE 2:** Fitting results of *in vitro* dissolution data for the first 20 min in the fasted and fed states.

States	Drug strengths	F_max_ (%)	k_d_(h^-1^)	β	t_lag_(h)
Fasted	10 mg	40.378	10.105	0.945	—
	20 mg	38.504	9.213	2.353	—
Fed	10 mg	64.200	0.494	1.340	0.0403
	20 mg	68.400	0.428	1.220	0.0411

#### 3.1.3 Validation of the fasted-state PBPK model

**FIGURE 2:**
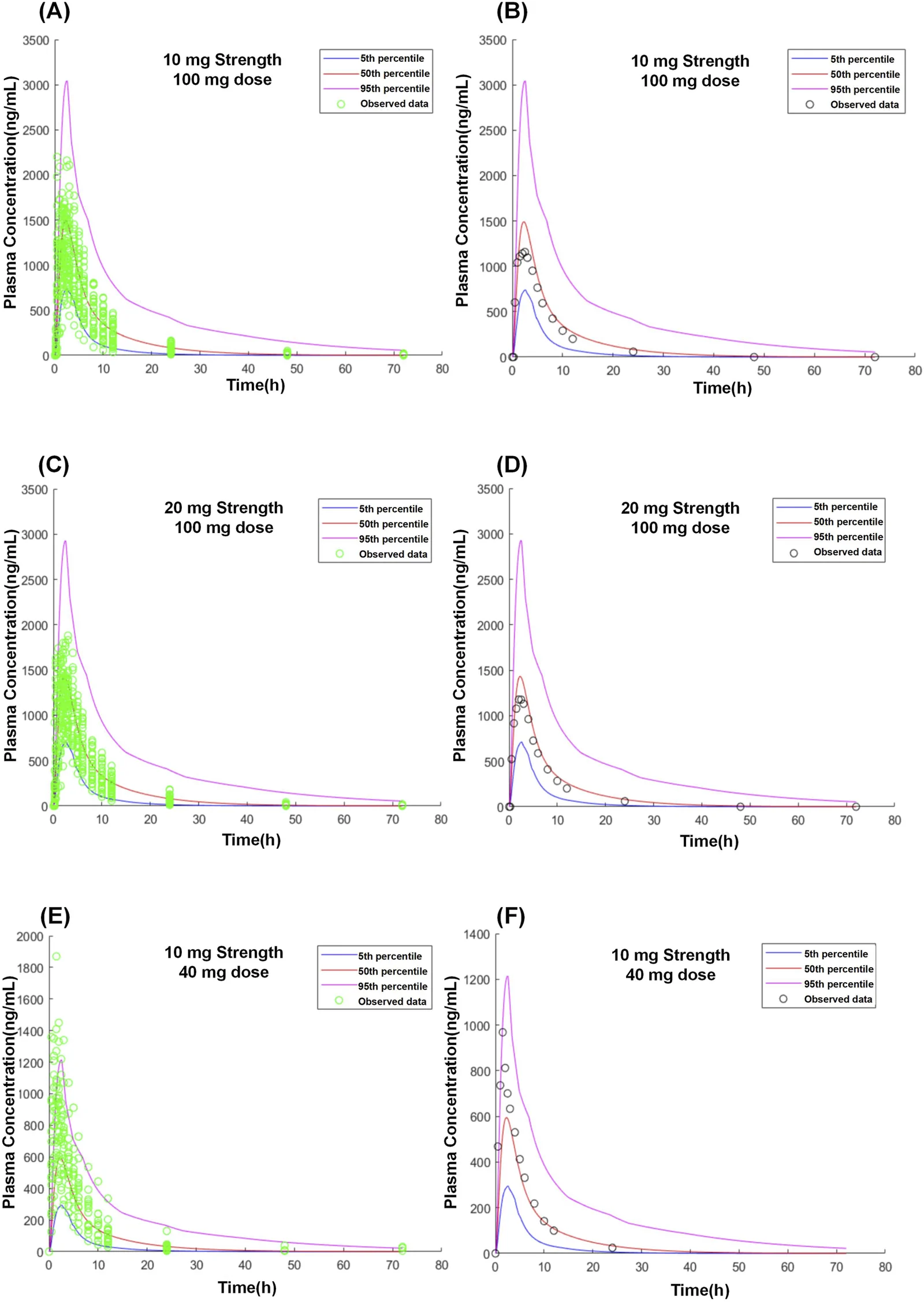
Comparison of simulated and observed PK profiles of senaparib capsules in the fasted state following single administration of 100 mg (IMP4297-110-1) and 40 mg (IMP4297-110-4) doses. The left panels **(A, C, E)** show individual observed PK data (scatter plots) versus simulated PK profiles (curves). The right panels **(B, D, F)** show the mean observed PK data (scatter plots) versus simulated PK profiles (curves).

The validation results in the fasted state were shown in Figure 2. For the 100 mg dose (Figures 2A-D), the majority of individual observed data points fell within the 90% CIs of the simulated profiles (Figures 2A and C). Furthermore, the mean observed data closely aligned with the 50th percentile of the simulated data (Figures 2B and D), confirming successful establishment of the fasted-state PBPK model. This predictive capability extended to the lower dose of 40 mg (Figures 2E,F), where the simulated profile also showed strong agreement with the observed data from study IMP4297-104, thereby validating the model’s accuracy within the linear dose range. Moreover, as shown in Supplementary Table S4, the Pred/Obs ratios for C_max_ and AUC_0-last_ were all within the 0.5- to 2-fold acceptance range.

**FIGURE 3:**
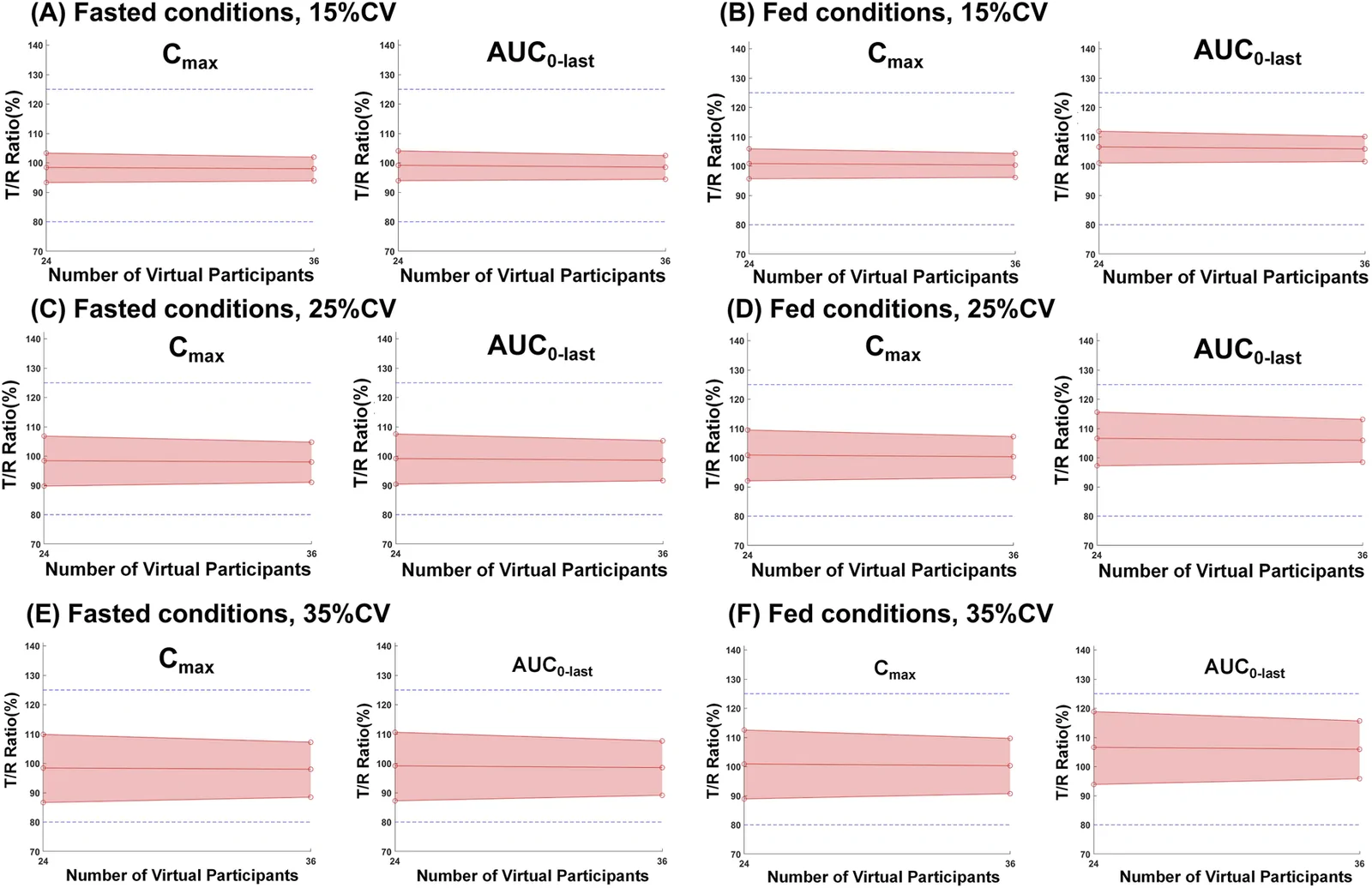
VBE simulation results for 10 mg and 20 mg senaparib capsules at a 100 mg dose under fasted and fed conditions with different intra-individual CVs. Panels **(A)**, **(C)**, and **(E)** show the fasted-state simulations with CVs of 15%, 25%, and 35%, respectively, whereas panels **(B)**, **(D)**, and **(F)** show the corresponding fed-state simulations. Red dots represent GMRs, and the shaded pink areas represent the 90% CIs. The upper and lower blue dashed lines denote the 80–125% bioequivalence acceptance limits. T and R denote the test (20 mg capsule) and reference (10 mg capsule) formulations of senaparib, respectively.

Subsequently, the VBE analysis was performed based on the simulated PK results and validated against the observed data from study IMP4297-110-1. The VBE results in the fasted state were shown in Figures 3A,C,E, with the corresponding simulation data summarized in Supplementary Table S5. The results demonstrated that the GMRs of Test/Reference for C_max_ and AUC_0-last_ were close to 100%, and the 90% CIs fell entirely within the 80%–125% range. This indicated that the test formulation was bioequivalent to the reference formulation at a 100 mg dose in the fasted state, which is consistent with observed results from IMP4297-110-1.

#### 3.1.4 Sensitivity analysis of the fasted-state PBPK model

The results of the sensitivity analysis were presented in Supplementary Figure S5; Supplementary Table S6. Overall, the predicted exposures of senaparib were relatively robust to the ±10% parameter perturbations, with maximum changes in these exposures not exceeding 10.3%. C_max_ was most sensitive to intestinal solubility, V, and CL, whereas AUC_0-inf_ was primarily affected by CL and intestinal solubility. Moreover, P_eff_ and gastric solubility showed a moderate influence on both C_max_ and AUC_0-inf_, whereas changes in V had a negligible impact on AUC_0-inf_.

### 3.2 Development and validation of the fed-state PBPK model

#### 3.2.1 Establishment of the *in vitro*-*in vivo* correlation in the fed state

Furthermore, the *in vivo* release profiles for the two formulations in the fed state were also fitted (Supplementary Table S7), with the corresponding *in vitro* dissolution profiles shown in Supplementary Table S3; Supplementary Figure S6. Comparison of the *in vitro* dissolution and the fitted *in vivo* release profiles in the fed state (Supplementary Figure S7) showed that the *in vitro* dissolution curves reached maximum dissolution within the first 20 min and exhibited a strong linear correlation with *in vivo* release (*R* ^2^ = 0.8263). However, the overall *in vivo* release rate was significantly slower than the *in vitro* dissolution rate, which is likely due to the inability of FeSSIF to adequately replicate the viscosity effects of food in the gastrointestinal tract.

Based on these findings, Weibull function parameters were estimated using the first 20 min of *in vitro* dissolution data and the resulting curves aligned well with the observed data (Supplementary Figures S4C,D). Subsequently, the model incorporated a *t*_*lag*_ to account for food-induced delayed gastric emptying and a 23.5-fold reduction in *k*_*d*_ to match the slower *in vivo* release in the fed state (Table 2). The *k*_*d*_-adjusted curves showed good agreement with the observed profiles, providing an accurate characterization of the *in vivo* release profiles in the fed state (Supplementary Figure S8).

#### 3.2.2 Validation of the fed-state PBPK model

The predictive performance of the fed-state PBPK model was also validated. As illustrated in Supplementary Figure S9, the majority of individual PK data fell within the 90% CIs of the simulated profiles and the mean observed data closely aligned with the 50th percentile. As shown in Supplementary Table S8, the Pred/Obs ratios for C_max_ and AUC_0-last_ were all within the 0.5- to 2-fold acceptance range. Moreover, VBE analysis further indicated that the GMRs for C_max_ and AUC_0-last_ were close to 100%, and the 90% CIs fell entirely within the 80%–125% range (Figures 3B,D,F; Supplementary Table S5). These results were consistent with the observed data from study IMP4297-110-2.

### 3.3 VBE analysis across the linear dose range

#### 3.3.1 VBE analysis in the fasted state

**FIGURE 4:**
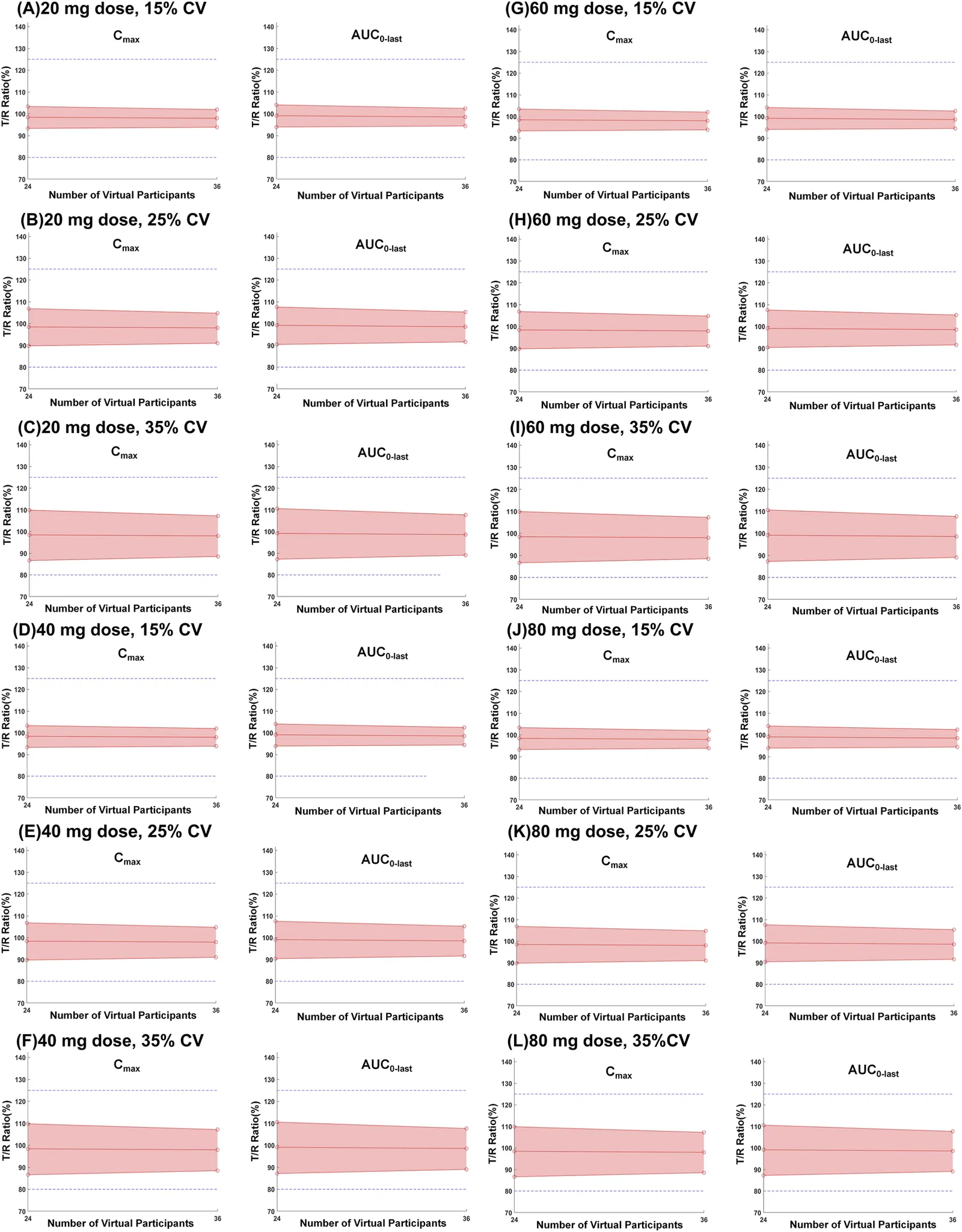
VBE simulation results for 10 mg and 20 mg senaparib capsules across the linear dose range (20–80 mg) under fasted conditions. The simulation scenarios incorporated sample sizes of 24 and 36 and intra-individual CVs of 15%, 25%, and 35%. Panels **(A–C)**, **(D–F)**, **(G–I)**, and **(J–L)** show the simulations at doses of 20, 40, 60, and 80 mg, respectively; within each dose group, the panels correspond sequentially to CVs of 15%, 25%, and 35%. Red dots represent GMRs, and the shaded pink areas represent the 90% CIs. The upper and lower blue dashed lines denote the 80–125% bioequivalence acceptance limits. T and R denote the test (20 mg capsule) and reference (10 mg capsule) formulations of senaparib, respectively.

Following the model validation, VBE was assessed for the 10 mg and 20 mg senaparib capsules. Simulations were performed across the linear dose range (20–80 mg) under various scenarios, including different sample sizes (n = 24, 36) and intra-individual CVs (15%, 25%, and 35%). As depicted in Figure 4; Table 3, the GMRs for C_max_ and AUC_0-last_ were close to 100%, and the 90% CIs fell entirely within the 80%–125% acceptance range. Collectively, these results supported the conclusion of bioequivalence between the two strengths across the linear dose range in the fasted state.

**TABLE 3:** Summary of VBE simulation results for Senaparib capsules following single administration of 20∼80 mg in the fasted state.

​		The T/R ratio of C_max_ and its 90% CI		The T/R ratio of AUC_0-last_ and its 90% CI	
Dose (mg)	Intra-individual CV (%)	Sample size N = 24	Sample size N = 36	Sample size N = 24	Sample size N = 36
20	15	98.428 (93.286∼103.386)	97.977 (93.856∼101.967)	99.133 (93.948∼104.089)	98.524 (94.405∼102.509)
20	25	98.402 (89.737∼106.831)	97.988 (90.981∼104.758)	99.124 (90.355∼107.526)	98.540 (91.579∼105.228)
20	35	98.420 (86.568∼109.850)	97.968 (88.463∼107.164)	99.115 (87.192∼110.542)	98.520 (89.062∼107.641)
40	15	98.391 (93.296∼103.361)	97.957 (93.844∼101.990)	99.099 (93.922∼104.068)	98.547 (94.393∼102.475)
40	25	98.386 (89.717∼106.826)	97.958 (90.966∼104.759)	99.123 (90.354∼107.549)	98.516 (91.553∼105.238)
40	35	98.410 (86.605∼109.812)	97.967 (88.447∼107.169)	99.122 (87.169∼110.572)	98.550 (89.048∼107.663)
60	15	98.476 (93.324∼103.427)	98.039 (93.863∼102.022)	99.174 (93.998∼104.156)	98.601 (94.457∼102.523)
60	25	98.404 (89.732∼106.815)	97.941 (90.977∼104.759)	99.127 (90.342∼107.512)	98.520 (91.545∼105.242)
60	35	98.475 (86.623∼109.883)	97.999 (88.477∼107.223)	99.152 (87.241∼110.616)	98.580 (89.071∼107.676)
80	15	98.414 (93.277∼103.375)	97.940 (93.842∼101.964)	99.115 (93.921∼104.101)	98.538 (94.404∼102.468)
80	25	98.466 (89.771∼106.842)	98.019 (91.000∼104.786)	99.188 (90.423∼107.591)	98.574 (91.589∼105.305)
80	35	98.454 (86.620∼109.878)	97.992 (88.504∼107.233)	99.155 (87.242∼110.597)	98.590 (89.094∼107.698)

T and R denote the test (20 mg capsule) and reference (10 mg capsule) formulations of senaparib, respectively.

#### 3.3.2 VBE analysis in the fed state

In the fed state, a VBE analysis was performed across the linear dose range (20–80 mg), employing the identical sample sizes and intra-individual CVs as those applied in the fasted-state analysis. As presented in Supplementary Figure S10; Supplementary Table S9, the GMRs for C_max_ and AUC_0-last_ were close to 100%, with the 90% CIs entirely within the 80%–125% acceptance range, thus establishing bioequivalence between the two strengths in the fed state.

## 4 Discussion

Senaparib (Sepalna®) is a novel PARP inhibitor being developed by IMPACT Therapeutics for the treatment of advanced epithelial ovarian, fallopian tube, or primary peritoneal cancer. Although bioequivalence between the 10 mg and 20 mg senaparib capsules has been established at the clinically recommended dose (100 mg), this conclusion cannot be reliably extrapolated to lower adjusted doses (e.g., 40 mg, 60 mg, and 80 mg) due to its nonlinear pharmacokinetics.

Currently, the recommendations on dose selection for BE studies of nonlinear PK compounds have not been specifically described in ICH M13A. In this context, the emergence of the VBE approach provides a potential and powerful tool to address this scientific gap. This approach combines PBPK modeling and *in vitro* dissolution data, allowing for the estimation of BE success rates in virtual populations across various trial designs. In this study, we conducted the VBE analysis to address the BE challenge posed by senaparib. The simulation results demonstrated the 10 mg senaparib capsules were bioequivalent to the 20 mg across the linear dose range (20–80 mg) under various scenarios, including different sample sizes (n = 24, 36), levels of intra-individual variability (15%, 25%, and 35%), and fasted/fed states.

During PBPK model development, permeation, distribution, and elimination parameters were assumed to be invariant across capsule strengths, doses and prandial states, with all *in vivo* PK variability attributed solely to differences in dissolution. This assumption was based on the following reasons: First, the BE study focused on the release of a drug substance from a drug product and its subsequent absorption into the systemic circulation. Therefore, the APIs from the test and reference products were considered to have no difference in disposition process. Second, the 10 mg and 20 mg senaparib capsules contained the same API and shared an identical formulation. Both formulations were manufactured using the same senaparib solid dispersion and pharmaceutical excipients via a direct powder filling process. Third, the similar PK profiles of the 10 mg and 20 mg senaparib capsules in studies IMP4297-110-1 and IMP4297-110-2 indicated that distribution and elimination were consistent across strengths, suggesting that observed PK differences were primarily due to variations in *in vivo* release. Consequently, the validated parameters listed in Table 1 were applied throughout the VBE analysis workflow. Nevertheless, this assumption represents a simplification of the physiological processes. Potential dose- or prandial-dependent changes in gastrointestinal physiology and absorption processes may not be fully captured.

The STS approach was selected for parameter estimation in the present PBPK-VBE analysis. This approach first estimated individual PK parameters from densely sampled clinical data, then derived population typical values and inter-individual variability, which were key inputs for virtual population generation in VBE simulations. STS provided a computationally efficient and transparent framework that was well suited for the extensive scenario-based VBE simulations performed in this study.

The establishment of IVIVC was critical to realize the prediction of *in vivo* release by *in vitro* pharmaceutical tools (Suarez-Sharp et al., 2018; Heimbach et al., 2019). First, it was a critical challenge to select the *in vitro* dissolution media that could mimic the *in vivo* dissolution behavior of the drug in the GI tract (Zhang et al., 2023). Given the low solubility of senaparib, its gastrointestinal absorption may be potentially influenced by physiological factors affecting drug solubilization and dissolution, such as the concentrations of solubilizing components (e.g., bile acids, bile salts, and phospholipids) and gastrointestinal pH conditions. Biorelevant media—FaSSIF (pH = 6.5) and FeSSIF (pH = 5.0)—were selected to mimic the physiological dissolution environment. To ensure consistent dissolution environments between the 10 mg and 20 mg senaparib capsules, dissolution profiles were compared using equivalent doses (one 20 mg capsule and two 10 mg capsules) in the identical biorelevant media. Second, deconvolution was applied to clinical PK data to precisely determine the *in vivo* release profile for establishing the IVIVC, thereby isolating the dissolution process from subsequent permeation and distribution (Margolskee et al., 2016). *In vivo* release showed a good linear relationship with *in vitro* dissolution within the first 20 min in the fasted and fed states. The Weibull function curve, based on the first 20 min of *in vitro* dissolution data in the fasted state, was used to characterize *in vivo* release. Notably, since FeSSIF incompletely replicated the viscosity effects of food in the GI tract, accurate prediction of the *in vivo* release was achieved by incorporating a *t*_*lag*_ and a 23.5-fold reduction in *k*_*d*_ parameter. This indicates that the fed-state model represents a clinically calibrated PBPK model incorporating empirical adjustments rather than a fully mechanistic prediction of food effects. Furthermore, this study lacked low-dose PK data in the fed state for model validation. More importantly, because senaparib is indicated for fasting administration, dosing in the fed state is generally not required in routine clinical use. Therefore, the fed-state VBE analysis across the 20–80 mg dose range was exploratory only.

Intra-subject variability is a critical factor in bioequivalence evaluation (Bego et al., 2022). In this study, simulations incorporated a range of intra-individual variability (15%, 25%, and 35%) that extended beyond the clinical observed range in IMP4297-110-1 and IMP4297-110-2 (15.4%–21.3%). Crucially, even under the most stringent condition of high variability (35%) coupled with a small sample size (n = 24), the 90% CIs for both C_max_ and AUC_0-last_ remained entirely within the 80%–125% BE range at all tested doses (20–80 mg). This demonstrated a key advantage of the VBE analysis: it established that the BE conclusion was not only statistically significant but also remarkably robust across a wide range of intra-individual variability, providing a depth of evidence that is hard to generate by a traditional BE study alone.

An additional observation from the VBE simulations is that the GMRs remained constant across tested doses, sample sizes, and intra-individual CV levels. This stability can be primarily attributed to the high similarity between the two capsule strengths, which exhibited nearly identical *in vitro* dissolution profiles and resulted in similar predicted *in vivo* release profiles through the established IVIVC relationship. In addition, the same systemic disposition parameters were applied for both formulations, further supporting comparable relative exposure between the test and reference products. The impact of sample size and intra-individual variability was primarily reflected in the width of the 90% CIs rather than the point estimates, which is consistent with statistical expectations for BE assessment.

In the context of the intended regulatory application, model qualification was supported by multiple lines of evidence, including adequate description of observed clinical PK profiles, prediction of an independent 40 mg PK dataset, and consistency between simulated and observed BE outcomes at the 100 mg dose. These evaluations supported the applicability of the PBPK-VBE framework for formulation bridging between the 10 mg and 20 mg senaparib capsule strengths within the evaluated dose range.

Several limitations should be acknowledged. First, the present model did not explicitly incorporate saturable absorption, transporter-mediated processes, precipitation, or nonlinear first-pass metabolism. Therefore, it should not be interpreted as a mechanistic explanation of the high-dose exposure plateau observed with senaparib. Instead, its application was restricted to the 20–80 mg linear PK range for the purpose of formulation bridging. Second, uncertainty in the estimated PBPK parameters was not explicitly propagated into the VBE simulations; this could be further addressed using formal uncertainty quantification approaches in future studies. Third, this study represented a single-compound case study, and the applicability of the PBPK-VBE approach to other drugs with dose-dependent or nonlinear pharmacokinetics requires further evaluation. Additional case studies and regulatory experience are needed to determine the broader utility of this strategy for formulation bridging.

Our study successfully demonstrated the application of a PBPK-VBE approach to support formulation bridging and BE risk assessment for senaparib, a drug exhibiting nonlinear pharmacokinetics. Based on this case study, we propose a prospective PBPK-VBE framework for new drugs with nonlinear pharmacokinetics—conducting a single BE study at a critical dose level to acquire data for PBPK model development and validation. Once appropriately qualified, the PBPK model—incorporating *in vitro* dissolution data—may provide supportive evidence for BE assessment at additional dose levels and facilitate more efficient drug development strategies.

## 5 Conclusion

In conclusion, this study successfully established a PBPK-VBE framework to bridge the bioequivalence of senaparib capsule strengths (10 mg and 20 mg) across the linear dose range, effectively addressing a key regulatory challenge posed by its nonlinear pharmacokinetics. This case study illustrated the potential utility of PBPK-VBE modeling as supportive evidence for formulation bridging, particularly when direct clinical BE studies across all dose levels are impractical.

## Data Availability

The original contributions presented in the study are included in the article/Supplementary Material, further inquiries can be directed to the corresponding author.
