# Supplementary material for: A PBPK-VBE approach for bioequivalence extrapolation of senaparib capsule strengths in the context of nonlinear pharmacokinetics: bridging from the clinical starting dose to the linear pharmacokinetic range
Source: Front Pharmacol. 2026 Sep 4;17:1918344. doi: 10.3389/fphar.2026.1918344 (PMC13585939; doi:10.3389/fphar.2026.1918344)
Supplement: Supplementary file 1 [file DataSheet1.ZIP › Supplementary Material/Supplementary_Material.docx]

Supplementary Material

## Supplementary Text S1. The details of the clinical studies

IMP4297-104 was a Phase I, open-label, fixed-sequence study conducted in healthy participants to evaluate the effects of multiple-dose itraconazole or rifampicin on the PK profile of a single-dose senaparib capsule. This trial (NMPA registration number: CTR20201970) was approved by the Medical Ethics Committee of Xiangya Hospital, Central South University (Hunan, China), and strictly adhered to the ethical principles of the Declaration of Helsinki and Good Clinical Practice (GCP). Written informed consent was obtained from all participants prior to enrollment. The study enrolled 32 healthy male participants aged 18–55 years, divided into two parallel cohorts (itraconazole and rifampicin groups). Participants received senaparib monotherapy in Period 1 at doses of 40 mg (10 mg capsule×4) for the itraconazole cohort or 100 mg (10 mg capsule×10) for the rifampicin cohort, followed by Period 2 treatment with the same senaparib dose co-administered with itraconazole 200 mg or rifampicin 600 mg, respectively. Blood samples were collected at −1h(pre-dose) and at 0.5, 1, 1.5, 2, 2.5, 3, 4, 5, 6, 8, 10, 12, 24, 48, 72, and 96h(post-dose) during both treatment periods. Plasma concentrations of senaparib were quantified using a validated liquid chromatography-tandem mass spectrometry (LC-MS/MS) method. Pharmacokinetic parameters were derived via non-compartmental analysis (NCA) with Phoenix WinNonlin® software (version 8.2; Certara USA, Princeton, NJ, USA)

IMP4297-110-1 and IMP4297-110-2 were randomized, single-dose, two-period, two-sequence crossover bioequivalence studies conducted in healthy Chinese participants to compare the pharmacokinetics of two capsule formulations (10 mg and 20 mg) of senaparib administered as a 100 mg dose in the fasted or fed states, respectively. These trials (China NMPA registration numbers: CTR20221034 and CTR20221035) were approved by the Biomedical Research Ethics Committee of Xuzhou Central Hospital (Jiangsu, China) and strictly adhered to the ethical principles of the Declaration of Helsinki and Good Clinical Practice (GCP). Written informed consent was obtained from all participants prior to enrollment. IMP4297-110-1 enrolled 28 healthy male participants aged 18–55 years, while IMP4297-110-2 enrolled 36 participants of the same demographic. Participants were randomized into two cohorts and administered either the test formulation (100 mg senaparib via 20 mg capsule×5) or the reference formulation (100 mg senaparib via 10 mg capsule×10) in two periods, separated by a 7-day washout period. In IMP4297-110-1(fasted-state), participants fasted overnight for at least 10 hours before dosing on Day 1, with no water intake permitted 1h pre-dose to 1h post-dose. Food intake was restricted until 4h post-dose. Serial blood samples were collected at −1h(pre-dose) and 0.25, 0.5, 1, 1.5, 2, 2.5, 3, 4, 5, 6, 8, 10, 12, 24, 48, and 72 h(post-dose). In IMP4297-110-2(fed-state), participants fasted overnight for at least 10 hours and consumed a standard high-fat breakfast starting 30 minutes before dosing. Water intake was restricted 1h pre-dose to 1h post-dose, with food permitted at least 4h post-dose. Serial blood samples were collected at −1 h (pre-dose) and 1, 2, 3, 4, 4.5, 5, 5.5, 6, 7, 8, 10, 12, 24, 48, and 72 h (post-dose). Plasma concentrations of senaparib were quantified using a validated high performance liquid chromatography-tandem mass spectrometry (HPLC-MS/MS) method, and pharmacokinetic parameters were derived via non-compartmental analysis (NCA). IF the 90% confidence intervals (CIs) of the maximum plasma concentration (C_max_), the area under the curve from time zero to time t (AUC_0-t_), the AUC from time zero to infinity (AUC_0-inf_) geomean ratio (GMR) fell within the acceptance range (80.00%-125.00%), the test formulation could be considered as bioequivalent to reference formulation.

## Supplementary Text S2. Detailed methodology for virtual bioequivalence simulations

To ensure rigorous reproducibility, the Mersenne Twister algorithm was used as the random number generator and initialized with a fixed seed 227 at the start of the simulation. All subsequent random sampling were deterministically derived from this single generator state. Virtual subjects were generated using stratified Monte Carlo sampling. First, baseline physiological parameters (sex, age, height, and body weight) were drawn from demographic distributions, with subjects falling outside the predefined body weight range being strictly excluded. Sampling continued from the same random sequence until the target sample sizes and sex ratios were met. For each virtual subject, V and CL were independently sampled from predefined normal distributions, and typical population values were scaled to individual values via allometric relationship. For each simulation scenario, 10 replicate virtual BE trials were performed using sample sizes of 24 and 36 virtual subjects per treatment arm. Each replicate simulation followed a standard randomized two-period, two-sequence crossover BE design, in which each virtual subject received both the test and reference formulations. Intra-subject variability was introduced post-simulation via an independent Bootstrap resampling approach. Specifically, the individual AUC and C_max_ values were used as a baseline, and random variability was introduced between treatment periods to represent within-subject exposure variability in the crossover design. This procedure mimics the random variations in PK parameters that the same subject may exhibit in the second period. The individual ratios of AUC and Cₘₐₓ were log-transformed, and the GMR was computed as the back-transformed mean of the log-ratios. The 90% CI for the GMR was constructed using the t-distribution:

$$\text{90\% CI = exp}\left( \bar{\ln R}\text{±}t_{\text{0.05,n}\text{-}\text{1}}\text{·}\frac{s_{\text{lnR}}}{\sqrt{n}} \right)$$

where ln R denotes the sample mean of the log-transformed individual ratios, ^S^ln R is the sample standard deviation, n is the number of virtual subjects, and t₀.₀₅,ₙ₋₁ is the two-sided critical value of the t-distribution at α = 0.10 with n − 1 degrees of freedom.

**Supplementary Table S1.** **Bioequivalence results between the 10 mg and 20 mg formulations following administration of a 100 mg dose in healthy participants in the fasted (IMP4297-110-1) and fed states (IMP4297-110-2)**

|  | **PK parameters** | **Geometric Mean Ratio (%)** | **90% Confidence Interval** | **Intra-individual CV (%)** | **Prandial states** | **Dose**  **(mg)** |
| --- | --- | --- | --- | --- | --- | --- |
| IMP4297-110-1 | C_max_ (ng/mL) | 101.82 | (93.89, 110.42) | 17.9209 | Fasted | 100 |
|  | AUC_0-last_ (ng·h/mL) | 97.91 | (89.91, 106.63) | 18.8795 | Fasted | 100 |
|  | AUC_0-inf_ (ng·h/mL) | 97.56 | (89.77, 106.02) | 18.4077 | Fasted | 100 |
| IMP4297-110-2 | C_max_ (ng/mL) | 108.85 | (99.53, 119.04) | 21.2739 | Fed | 100 |
|  | AUC_0-last_ (ng·h/mL) | 105.14 | (98.27, 112.50) | 15.8627 | Fed | 100 |
|  | AUC_0-inf_ (ng·h/mL) | 105.63 | (98.93, 112.77) | 15.3549 | Fed | 100 |

**Supplementary Table S2.** **Fitted *in vivo* release results of 10mg and 20mg senaparib capsules in the fasted state**

| **Strengths** | **Fmax (%)** | **kd (h-1)** | **β** |
| --- | --- | --- | --- |
| 10mg | 40.100 | 9.050 | 1.610 |
| 20mg | 40.400 | 10.100 | 0.945 |

**Supplementary Table S3.** ***In vitro* dissolution data for 10mg and 20mg senaparib capsules in the fasted and fed states**

|  | **Dissolution rate (%)** | | | | | | | | | |
| --- | --- | --- | --- | --- | --- | --- | --- | --- | --- | --- |
| Time  (min) | 5 | 10 | 15 | 20 | 30 | 45 | 60 | 90 | 120 | 150 |
| 10mg capsule*2  (Fasted-State) | 16 | 36 | 39 | 38 | 37 | 33 | 27 | 20 | 16 | 13 |
| 20mg capsule*1  (Fasted-State) | 23 | 33 | 36 | 39 | 44 | 32 | 25 | 17 | 13 | 11 |
| 10mg capsule*2  (Fed-State) | 21 | 52 | 62 | 64 | 63 | 64 | 62 | 60 | 58 | 56 |
| 20mg capsule*1  (Fed-State) | 21 | 49 | 63 | 69 | 68 | 67 | 64 | 60 | 56 | 53 |

**Supplementary Table S4. Comparison of simulated and observed pharmacokinetic parameters of senaparib in the fasted state**

| **Doses** | **Strengths** | **PK Parameter**  **(N=36)** | **Predicted** | **Observed** | **Ratio(P/O)** | **Fold-Error** |
| --- | --- | --- | --- | --- | --- | --- |
| 40mg | 10mg | C_max_  (ng/mL) | 637.1 | 977 | 0.65 | 1.53 |
|  |  | AUC_0-last_ (ng/mL*h) | 6241 | 6187.7 | 1.01 | 1.01 |
| 100mg | 10mg | C_max_  (ng/mL) | 1591 | 1442.0 | 1.10 | 1.10 |
|  |  | AUC_0-last_ (ng/mL*h) | 15580 | 10767.4 | 1.45 | 1.45 |
|  | 20mg | C_max_  (ng/mL) | 1522 | 1329.1 | 1.15 | 1.15 |
|  |  | AUC_0-last_ (ng/mL*h) | 14910 | 10280.8 | 1.45 | 1.45 |

**Supplementary Table S5. Summary of VBE simulation results for senaparib at a 100 mg dose in the fasted and fed states**

|  |  | | **The T/R ratio of C_max_ and its 90% CI** | | **The T/R ratio of AUC_0-last_ and its 90% CI** | |
| --- | --- | --- | --- | --- | --- | --- |
| **Fasted-State** | **Dose**  **(mg)** | **Intra-individual CV (%)** | **sample size**  **N=24** | **sample size**  **N=36** | **sample size**  **N=24** | **sample size**  **N=36** |
|  | 100 | 15 | 98.406  (93.279~103.364) | 97.986  (93.844~101.959) | 99.147  (93.920~104.101) | 98.509  (94.395~102.494) |
|  | 100 | 25 | 98.438  (89.747~106.844) | 98.025  (91.030~104.782) | 99.186  (90.410~107.566) | 98.590  (91.600~105.259) |
|  | 100 | 35 | 98.460  (86.657~109.886) | 98.038  (88.474~107.237) | 99.189  (87.238~110.624) | 98.577  (89.107~107.668) |
| **Fed-State** | 100 | 15 | 100.843  (95.652~105.930) | 100.315  (96.123~104.389) | 106.563  (101.025~111.894) | 105.877  (101.517~110.121) |
|  | 100 | 25 | 100.936  (92.072~109.494) | 100.358  (93.231~107.247) | 106.640  (97.240~115.619) | 105.970  (98.492~113.116) |
|  | 100 | 35 | 100.916  (88.885~112.567) | 100.332  (90.688~109.702) | 106.634  (93.853~118.860) | 105.965  (95.805~115.686) |

**Supplementary Table S6. Sensitivity analysis of key PBPK parameters on senaparib Cₘₐₓ and AUC predictions**

| **Parameter** | **C_max_ Change (%)** | | **AUC Change (%)** | |
| --- | --- | --- | --- | --- |
|  | +10% | -10% | +10% | -10% |
| P_eff_ | 2.956 | -3.216 | 2.652 | -2.999 |
| Sol Stomach | 2.233 | -2.185 | 1.891 | -1.892 |
| Sol Intestine | 7.734 | -7.697 | 7.974 | -7.993 |
| V | -5.176 | 5.937 | -0.629 | 0.508 |
| CL | -4.218 | 4.762 | -8.667 | 10.327 |

**Supplementary Table S7.** **Fitted *in vivo* release results of 10mg and 20mg senaparib capsules in the fed state**

| **Strengths** | **Fmax (%)** | **kd(h-1)** | **β** | **tlag(h)** |
| --- | --- | --- | --- | --- |
| 10mg | 64.000 | 0.470 | 1.330 | 0.050 |
| 20mg | 66.000 | 0.482 | 1.350 | 0.050 |

**Supplementary Table S8. Comparison of simulated and observed pharmacokinetic parameters of senaparib in the fed state**

| **Doses** | **Strengths** | **PK Parameter**  **(N=36)** | **Predicted** | **Observed** | **Ratio(P/O)** | **Fold-Error** |
| --- | --- | --- | --- | --- | --- | --- |
| 100mg | 10mg | C_max_  (ng/mL) | 1845 | 1652.4 | 1.12 | 1.12 |
|  |  | AUC_0-last_ (ng/mL*h) | 21220 | 17180.88 | 1.24 | 1.24 |
|  | 20mg | C_max_  (ng/mL) | 1808 | 1912.1 | 0.95 | 1.06 |
|  |  | AUC_0-last_ (ng/mL*h) | 21800 | 19740.31 | 1.10 | 1.10 |

**Supplementary Table S9.** **Summary of VBE simulation results for senaparib capsules following administration of 20-80 mg in the fed state**

|  | | **The T/R ratio of C_max_ and its 90% CI** | | **The T/R ratio of AUC_0-last_ and its 90% CI** | |
| --- | --- | --- | --- | --- | --- |
| **Dose**  **(mg)** | **Intra-individual CV (%)** | **sample size**  **N=24** | **sample size**  **N=36** | **sample size**  **N=24** | **sample size**  **N=36** |
| 20 | 15 | 100.901  (95.681~105.977) | 100.366  (96.150~104.442) | 106.629  (101.076~111.915) | 105.954  (101.537~110.149) |
| 20 | 25 | 100.849  (92.005~109.420) | 100.308  (93.164~107.217) | 106.568  (97.214~115.570) | 105.873  (98.469~113.042) |
| 20 | 35 | 100.872  (88.801~112.495) | 100.285  (90.644~109.654) | 106.560  (93.838~118.813) | 105.875  (95.771~115.608) |
| 40 | 15 | 100.855  (95.656~105.930) | 100.273  (96.118~104.379) | 106.575  (101.039~111.855) | 105.917  (101.509~110.093) |
| 40 | 25 | 100.889  (92.054~109.495) | 100.336  (93.256~107.232) | 106.647  (97.268~115.625) | 105.965  (98.478~113.072) |
| 40 | 35 | 100.892  (88.861~112.547) | 100.365  (90.681~109.744) | 106.612  (93.865~118.847) | 105.963  (95.803~115.664) |
| 60 | 15 | 100.900  (95.686~105.989) | 100.336  (96.167~104.401) | 106.609  (101.103~111.954) | 105.967  (94.457~102.523) |
| 60 | 25 | 100.902  (92.039~109.463) | 100.329  (93.237~107.220) | 106.632  (97.223~115.595) | 105.967  (98.500~113.100) |
| 60 | 35 | 100.852  (88.838~112.526) | 100.297  (90.641~109.670) | 106.552  (93.836~118.798) | 105.908  (95.763~115.630) |
| 80 | 15 | 100.862  (95.657~105.935) | 100.274  (96.107~104.384) | 106.594  (101.041~111.877) | 105.873  (101.499~110.093) |
| 80 | 25 | 100.883  (92.005~109.399) | 100.312  (93.171~107.214) | 106.594  (97.198~115.545) | 105.905  (98.468~113.021) |
| 80 | 35 | 100.901  (88.885~112.558) | 100.371  (90.653~109.743) | 106.616  (93.882~118.857) | 105.954  (95.804 ~115.663) |

Note: T and R denote the test (20 mg capsule) and reference (10 mg capsule) formulations of senaparib, respectively.

**Supplementary Figure S1. Comparison of simulated and observed PK profiles of 10mg and 20mg senaparib capsules following administration of a 100 mg dose in study IMP4297-110-1.** (A)Individual observed PK data (scatter plots) versus simulated PK profiles (curves). (B) Mean observed PK data (scatter plots) versus simulated PK profiles (curves).

**Supplementary Figure S2. Comparison of *in vitro* dissolution profiles between 10 mg and 20 mg senaparib capsules in the fasted state**

**Supplementary Figure S3. Comparison of *in vitro* dissolution and *in vivo* release profiles of 10 mg and 20 mg senaparib capsules in the fasted state**

**Supplementary Figure S4. Comparison of observed dissolution data (Scatter plots) and Weibull model-simulated profiles (Curves) in the fasted and fed states during the first 20 minutes.**

(A) 10 mg strength in the fasted state; (B) 20 mg strength in the fasted state;

(C) 10 mg strength in the fed state; (D) 20 mg strength in the fed state.

**Supplementary Figure S5.** Sensitivity analysis of key PBPK parameters on senaparib Cₘₐₓ and AUC predictions

**Supplementary Figure S6. Comparison of *in vitro* dissolution profiles between 10 mg and 20 mg senaparib capsules in the fed state**

**Supplementary Figure S7. Comparison of *in vitro* dissolution and *in vivo* release profiles of 10 mg and 20 mg senaparib capsules in the fed state**

**Supplementary Figure S8. Weibull-based *in vivo* release curves in the fed state after optimization of the *k_d_* parameter**

**Supplementary Figure S9. Comparison of simulated and observed PK profiles of senaparib capsules in the fed state following administration of 100 mg (IMP4297-110-2).** The left panels (A and C) show individual observed PK data (scatter plots) versus simulated PK profiles (curves). The right panels (B and D) show the mean observed PK data (scatter plots) versus simulated PK profiles (curves)

**Supplementary Figure S10. VBE simulation results of 10 mg and 20 mg senaparib capsules across the linear dose range (20-80 mg) in the fed state.** The simulation scenarios incorporated sample sizes (n=24, 36) and intra-individual CVs (15%, 25%, 35%). Red dots represent geometric means; Shaded pink areas indicate 90% CIs. The upper and lower blue dashed lines denote the 80-125% bioequivalence acceptance criteria; T and R denote the test (20 mg capsule) and reference (10 mg capsule) formulations of senaparib, respectively
